# Striking Similarities in the Genomic Distribution of Tandemly Arrayed Genes in *Arabidopsis* and Rice

**DOI:** 10.1371/journal.pcbi.0020115

**Published:** 2006-09-01

**Authors:** Carene Rizzon, Loic Ponger, Brandon S Gaut

**Affiliations:** 1 Department of Ecology and Evolutionary Biology, University of California Irvine, Irvine, California, United States of America; 2 Department of Statistics and Genomics, Universite´ Evry Val d'Essonne, Evry, France; 3 Régulation et Dynamique des Génomes, Muséum National d'Histoire Naturelle, Paris, France; National Center for Genome Resources, United States of America

## Abstract

In *Arabidopsis,* tandemly arrayed genes (TAGs) comprise >10% of the genes in the genome. These duplicated genes represent a rich template for genetic innovation, but little is known of the evolutionary forces governing their generation and maintenance. Here we compare the organization and evolution of TAGs between *Arabidopsis* and rice, two plant genomes that diverged ~150 million years ago. TAGs from the two genomes are similar in a number of respects, including the proportion of genes that are tandemly arrayed, the number of genes within an array, the number of tandem arrays, and the dearth of TAGs relative to single copy genes in centromeric regions. Analysis of recombination rates along rice chromosomes confirms a positive correlation between the occurrence of TAGs and recombination rate, as found in *Arabidopsis.* TAGs are also biased functionally relative to duplicated, nontandemly arrayed genes. In both genomes, TAGs are enriched for genes that encode membrane proteins and function in “abiotic and biotic stress” but underrepresented for genes involved in transcription and DNA or RNA binding functions. We speculate that these observations reflect an evolutionary trend in which successful tandem duplication involves genes either at the end of biochemical pathways or in flexible steps in a pathway, for which fluctuation in copy number is unlikely to affect downstream genes. Despite differences in the age distribution of tandem arrays, the striking similarities between rice and *Arabidopsis* indicate similar mechanisms of TAG generation and maintenance.

## Synopsis

The nuclear genomes of higher plants vary tremendously in size and gene content. Much of this variation is attributable to gene duplication. To date, most studies of plant gene duplication have focused on whole genome duplication events, which duplicate all genes simultaneously. Another prominent process is single gene duplication, which often results in duplicated genes arranged in a tandem array. Here Rizzon, Ponger, and Gaut identify tandem arrays in rice and their genome organization between *Arabidopsis* and rice, two plant species that diverged ~150 million years ago. The two genomes contain a similar proportion of genes that are tandemly arrayed, with a similar number of genes within an array. Moreover, tandemly arrayed genes are most common in genomic regions of high recombination in both species. This organization appears to be a general feature of eukaryotic genomes, perhaps because duplication rates are higher in high recombination regions. Tandemly arrayed genes of rice and *Arabidopsis* also represent a biased gene set with regard to function. In contrast to genes duplicated through whole genome events, tandemly arrayed genes are enriched for genes that encode membrane proteins and genes that function in response to environmental stresses. Taken together, these observations suggest that tandemly arrayed genes represent a rich and relatively fluid source for plant adaptation.

## Introduction

The genomes of Arabidopsis thaliana (*Arabidopsis*) and Oryza sativa (rice) contain substantial proportions of duplicated chromosomal segments, presumably reflecting ancient polyploidy (paleopolyploid) events. In *Arabidopsis,* for example, there have been at least three paleopolyploid events [1], with the most recent occurring ~25 million years ago [2]. The duplicated chromosomal regions retain ~25% of their genes as duplicates [3], with the remaining duplicate pairs having lost one copy to deletion or pseudogenization. Surprisingly, the process of gene loss is nonrandom with respect to function, because genes that are retained as duplicates are enriched for functions related to transcription, signal transduction, and development [1,2]. Like *Arabidopsis,* rice also has a history of extensive duplication [4], with up to ~60% of the genome apparently duplicated by paleopolyploid events [5] and up to ~50% of genes retained as duplicates on duplicated chromosomal segments [6].

Although there have been numerous studies to identify genes duplicated via paleopolyploidy, one important source of duplication in plant genomes has not been studied in great detail: tandemly arrayed genes (TAGs). TAGs are gene family members that are tightly clustered on a chromosome, and they are frequent in plant genomes. In *A. thaliana,* TAGs comprise almost as many genes (up to 18%) as those duplicated by paleopolyploid events (~25%) [7]. They also represent a broad functional component of the genome, ranging from genes that encode secondary metabolites [8], to disease resistance genes [9], to regulatory genes [10].

The evolution and organization of TAGs have been studied in *Arabidopsis*. TAGs are underrepresented in centromeric regions relative to non-TAG genes, and their prevalence relative to non-TAG genes is positively correlated with recombination rates along chromosomes [11]. The evolutionary processes contributing to this correlation are unclear. The correlation could reflect the generation of TAGs via recombination-mediated processes such as unequal crossing-over (UCO), or it could be produced indirectly by interplay among selection, recombination, gene gain, and gene loss. It is also unclear whether the TAG organization in *Arabidopsis* is representative of other plant genomes.

TAGs are also likely to differ from dispersed (i.e., nonclustered) gene families in their process of divergence. The close physical proximity of TAGs facilitates gene conversion, as recently demonstrated in both yeast [12] and *Arabidopsis* [13]. One practical ramification is that the synonymous distance (Ks) between TAGs cannot be easily used as a proxy for the time of the duplication event that gave rise to the two genes [14]. Instead, Ks provides insight into *either* the age of the duplication event or the age of homogenizing gene conversion events [12]. Nonetheless, careful study of Ks values among clustered genes could uncover clues to TAG maintenance and diversification.

The completion of the rice genome sequence provides the first opportunity to compare the structure and evolution of TAGs between two plant genomes, *Arabidopsis* and rice. The two species diverged ~150 million years ago [15] but are similar in that they have relatively small genomes and reproduce predominantly by selfing. Genomic analyses of the rice sequence have already revealed some properties of TAGs—i.e., that TAGs compose between 16% [16] and 29% of rice genes [17] and that the preponderance of tandemly duplicated genes are differentiated by relatively low (<0.2) Ks values [16]. Nonetheless, TAGs in rice have not been studied in a comparative context nor in the context of genomic features such as chromosomal location and recombination.

In this paper, we address several basic questions about the organization, evolution, and function of TAGs. First, does the number and distribution of TAGs differ substantially between rice and *Arabidopsis*? Second, are TAGs more frequent in high recombination regions in rice, as they are in *Arabidopsis*? Third, do the two species exhibit clear similarities or differences in the distribution of Ks among TAGs? Fourth, do genes in TAGs represent functional biases relative to non-TAG genes? Finally, can we infer any general mechanisms that contribute to similarities and differences between the distribution of TAGs in the *Arabidopsis* and rice genomes?

## Results

### The Number, Size, and Physical Distribution of TAGs

The number of TAGs identified in *Arabidopsis* and rice depends on the TAG definition. The definition depends both on the criteria used to define homologous gene sets—of which TAGs are a subset—and the number of gene spacers allowed between TAGs. Because of this dependency on definition, we analyzed two groups of four datasets in both rice and *Arabidopsis*. The first group corresponds to a “low stringency” (L) criterion, corresponding to >30% identity and >70% alignment length, to identify sets of homologous genes. We refer to these homologous sets as superfamilies (see Materials and Methods). TAGs are physically adjacent genes within superfamilies. We defined four sets of TAGs corresponding to zero, one, five, and up to ten intervening spacer genes. The second group of datasets was based on high stringency (H) criteria (>50% identity and >90% alignment length), again with four spacer lengths. Hereafter, the datasets are named by stringency and spacer—e.g., the low stringency dataset with zero spacer genes is L/0.

Based on our analyses, genes within superfamilies compose from 40% to 67% of A. thaliana genes and 19% to 45% of O. sativa genes (Table 1). Both species contain substantial proportions of TAGs; up to 16% of *Arabidopsis* genes are TAGs, but the maximal proportion of rice genes is ~14%. These values are slightly lower than previous studies in *Arabidopsis* [11,18] and rice [16,17], because our definition of homology is more stringent. Rice also has a lower proportion of TAGs relative to *Arabidopsis* for all TAG definitions.

**Table 1:**
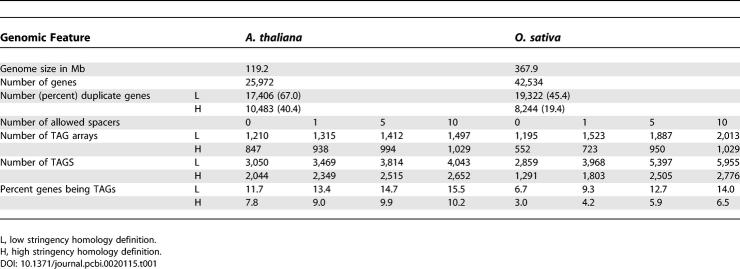
Identification of TAGs in the A. thaliana and O. sativa Genomes****

**Figure 1:**
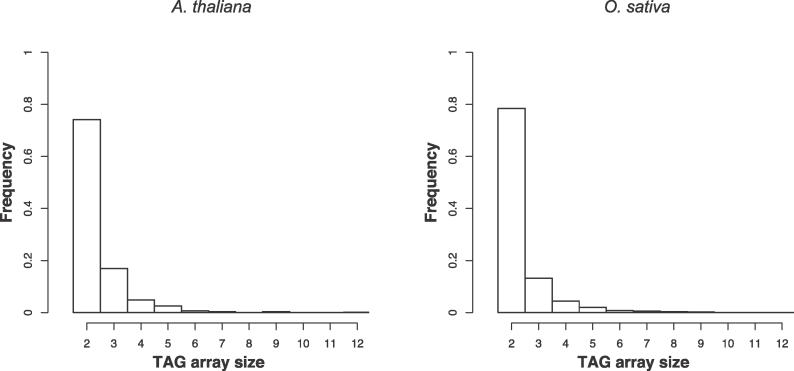
Distribution of the Size of TAGs for the H/0 Dataset (A) A. thaliana. (B) O. sativa.

For all datasets, most tandem arrays contained very few genes. For example, with the H/0 data set, 75% of *Arabidopsis* TAG arrays contained only two genes, and 79% of rice arrays contained only two genes (Figure 1; Table S1). The number of tandem arrays with three genes decreased sharply (to 17% of *Arabidopsis* and 14% in rice), and tandem arrays with more than three members were very rare (8% in *Arabidopsis,* with a maximum size of 12; 7% in rice, with a maximum size of nine). The size distributions of tandem arrays in rice and *Arabidopsis* are very similar (Figure 1) and statistically indistinguishable for several datasets (e.g., H/0: χ^2^ = 3.9, *p* = 0.14; H/10: χ^2^ = 1.81, *p* = 0.41), suggesting similar evolutionary constraints on array size in the two diverse lineages.

Both TAGs and non-TAG genes are physically clustered along each of the rice chromosomes (TAGs: maximum *p*-value of 12 tests = 0.0025; non-TAGs: maximum *p*-value of 12 tests = 1.49 × 10^–10^), even after Bonferroni correction for 12 tests. Both TAGs and non-TAGs have a strong tendency to cluster near the end of chromosomal arms (unpublished data).

### Estimated Recombination Rates (*ĉ*) along Rice Chromosomes and Correlation with TAG Density

**Figure 2:**
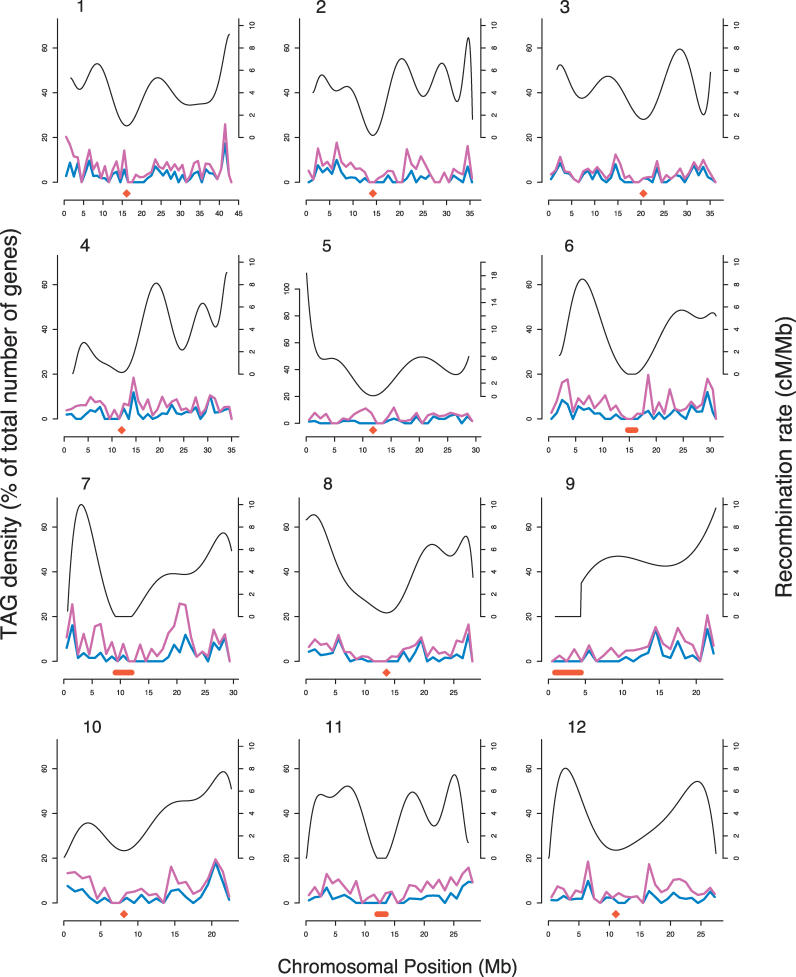
Recombination Rate Estimates and Density of TAGs (Number of TAGs/Total Number of Genes) along O. sativa Chromosome Recombination estimates are represented by the black lines. Density estimates are based on the H/0 dataset (blue lines) and H/10 dataset (pink lines). Centromere positions are marked in orange.

In *Arabidopsis,* there is a dearth of TAGs within pericentromeric regions, and the distribution of TAGs is positively correlated with recombination rate even when pericentromeric regions are removed from analysis [11]. To assess whether the rice genome is organized similarly, we first estimated recombination rates (*ĉ*) by comparing physical and genetic maps. Average *ĉ* ranged from 3.58 cM/Mb to 4.46 cM/Mb for all chromosomes, with an average of 4.12 cM/Mb across chromosomes (Table S2). Ignoring telomeres, where there may be some statistical artifact in estimation of *c,* most chromosomes had peak recombination rates ~9 cM/Mb and minimum rates approaching 0 cM/Mb. For several chromosomes (6, 7, 9, and 11), there was a pronounced region of low recombination, which we attributed to centromeric suppression and therefore defined as pericentromeric regions (Figure 2). Other chromosomes had less exaggerated regions of low recombination, and we thus defined centromeric regions on these chromosomes as low “points” of recombination rather than “regions.” Our resulting centromeric definitions corresponded closely to those reported previously [16,19]. Because most chromosomal arms had more than one apparent peak of recombination, a pattern that does not mimic other species closely, we also verified estimates of *ĉ* with an alternative method that considered chromosomal arms separately (see Materials and Methods). The two methods gave highly correlated results (Spearman rank correlation: for all 12 chromosomes *ρ* ≥ 0.70; *p* < 2.2 × 10^−16^).

We next determined if TAGs were underrepresented in centromeres. The physical distribution of TAGs relative to the total number of genes along O. sativa chromosomes is shown in Figure 2 for the H/0 and H/10 datasets. The distribution suggests a lower density of TAGs around centromeres compared with other chromosomal regions for a few chromosomes (1, 2, 3, 4, 6, 7, 8, and 11; see Figure 2). The trend is not readily obvious on all chromosomes, but we tested this more formally. We were able to define centromeres as *regions* (rather than single points) on chromosomes 6, 7, 9, and 11. When the data were combined across these four chromosomes, TAGs were significantly underrepresented in centromeres for TAG definitions L/0, L/1, L/5, L/10, H/5, and H/10 (χ^2^_minimum_ = 29.3, *p*_maximum_ = 6.1 × 10^−08^). We did the same analyses considering also 2-Mb regions around the “point” estimates for centromeres, and thereby combined information across all 12 chromosomes. TAGs were significantly underrepresented in centromeres relative to non-TAG genes for all eight TAG definitions (χ^2^_minimum_ = 30, *p*_maximum_ = 4.3 × 10^−08^). These results remained significant after Bonferroni correction. It is thus very clear that TAGs are underrepresented in centromeres relative to non-TAG genes in *O. sativa,* as they are in *A. thaliana*.

**Figure 3:**
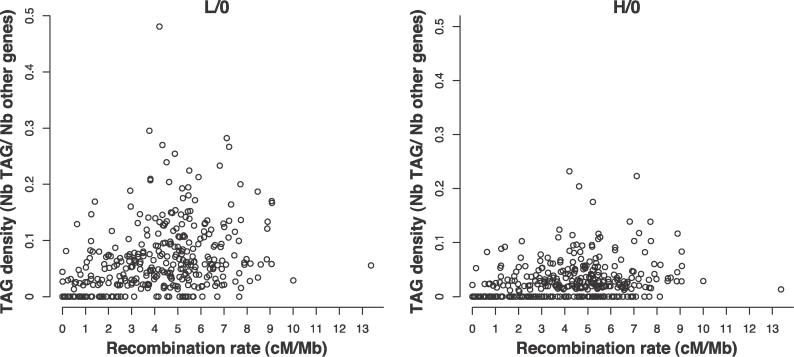
TAG Gene Density Plotted against Recombination Rate in O. sativa for the L/0 Dataset and the H/0 Dataset

Finally, we assessed the correlation between TAG density and *ĉ*. For five of 12 chromosomes tested separately (1, 6, 7, 8, and 11), correlations were positive and still significant after a Bonferroni correction for at least one of the eight TAG definitions (Table S3). The remaining chromosomes (3, 4, 5, 9, and 10) exhibited a range of correlations, both positive and negative, that were not statistically significant after Bonferroni correction (Table S3). However, when TAG density was plotted again for the entire rice genome (Figure 3), there was a positive but weak correlation for all eight TAG definitions (*p*_maximum_ = 7.7 × 10^−07^), with *ρ* ranging from 0.26 to 0.41 (Table 2). The correlation remained significant when regions with recombination estimates of 0.0 (i.e., pericentromeric regions and other low recombination regions) were removed from the analysis (*ρ* ranged 0.23 to 0.37 across TAG definitions, with *p*_maximum_ = 2.5 × 10^−05^). When regions lacking TAGs (i.e., regions with 0.0 values in the *y*-axis of Figure 3) were also removed from analysis, the correlation remained significant for six of eight TAG definitions (*ρ* ranged from 0.16 to 0.26; *p*_maximum_ = 0.007). We thus conclude that the density of TAGs is positively, but weakly, associated with recombination rate along rice chromosomes.

**Table 2:**
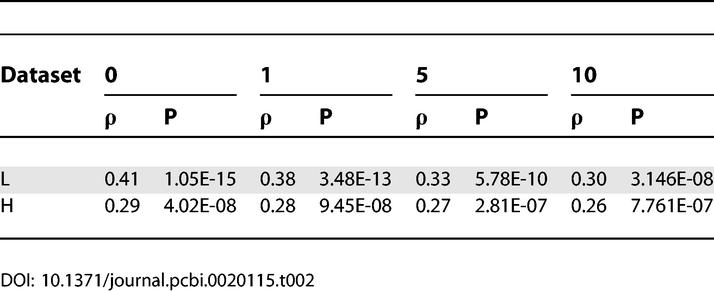
Spearman Rank Correlation Tests Comparing Recombination and Gene Density over All Chromosomes for Each Rice Dataset

### Pairwise Ks Distributions

Pairwise Ks distributions of duplicated genes have been used to infer the polyploid origin of plant genomes [2,6] and also to study the tempo and mode of gene duplication [14]. Here we compare the Ks distribution for TAGs and duplicated non-TAG genes between species, for two reasons. The first is to determine whether the pairwise Ks distribution for TAGs is similar between species. The second is to determine whether Ks is also correlated with *ĉ*_._

**Figure 4:**
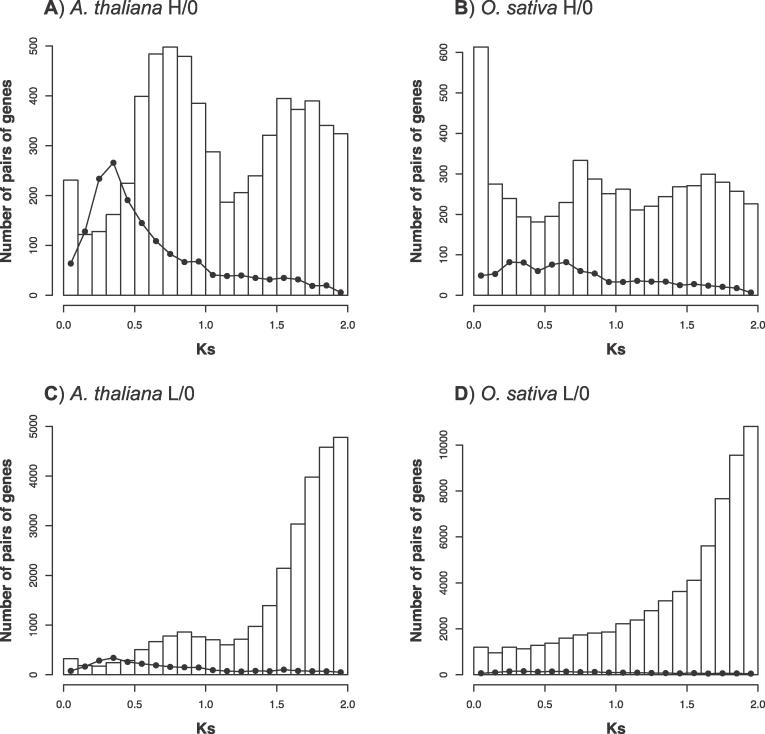
Distribution of Ks Values between TAG Pairs and between Duplicated Non-TAG Gene Pairs Histogram of the distribution of Ks values for TAG pairs (dots) and duplicated non-TAG gene pairs (bars) in *Arabidopsis* and in rice for the H/0 dataset are in panels (A) and (B), respectively. Results for the L/0 dataset are provided in panels (C) and (D).

In *Arabidopsis,* the pairwise Ks distribution for TAGs defines a clear peak around Ks ~ 0.3 in all of the L and H datasets (Figure 4A and 4C). The Ks distribution for duplicated non-TAG genes was markedly different but varied by dataset. In the H datasets (Figure 4A), the Ks distribution peaked in the range of 0.6 to 0.9 with another peak >1.5. With the L dataset, the Ks distribution shifted markedly toward higher Ks values (Figure 4C), reflecting less stringent definition of duplicates. These Ks peaks in duplicated non-TAG genes have been interpreted as evidence for ancient polyploid events [2,6]. The most notable feature of *Arabidopsis* Ks values is that the Ks peak for TAGs has a lower value than duplicated non-TAGs, as noted previously [2,20]. In contrast to *Arabidopsis,* the rice Ks distributions were not nearly as dramatic: across datasets, neither TAGs nor non-TAGs produced consistent Ks peaks (Figure 4).

Because the density of TAGs is correlated with recombination rate and because recombination could play a role in the production and divergence of TAGs, it is reasonable to try to assess whether Ks is correlated with *ĉ*. To assess this correlation, we focused on TAG arrays with only two genes, because the Ks distribution of TAGs with more than two members is biased toward overrepresentation of older pairs. We found no significant relationship between *ĉ* and Ks values for any dataset (Spearman rank Correlation, *ρ*_minimum_ = −0.08, *ρ*_maximum_ = 0.09, *ρ_minimum_* = 0.05), just as there was no correlation between molecular divergence and *ĉ* in *Arabidopsis* [11].

### TAG Functional Specificities

Previous studies have shown that genes retained as duplicates after polyploidy events represent a biased subset of molecular functions (MFs). To examine the functional specificities of TAGs, we identified Gene Ontology (GO) terms and compared TAGs with non-TAG (duplicated) genes and also with singleton genes that could not be assigned to a superfamily. For each term, we identified GO-slim terms in three categories: MF, biological process (BP), and cellular component (CC) [21] (see Materials and Methods). Our primary motivation for this analysis was to evaluate whether TAGs, like genes retained after polyploidy duplication, are biased toward particular functions.

For simplicity we explored the specificity of function for TAGs only for the H/0 (Table 3) and H/10 (Table S4) datasets. For A. thaliana, at least one GO term was associated with each gene. For TAGs, non-TAG genes, and singletons, in any of the MF, BP, and CC categories, at least 78% of the genes were linked to one or several GO terms. In *O. sativa,* we were able to associate at least one GO term to only ~68% (H/0 dataset) and ~66% (H/10 dataset) of the TAGs, to ~64% of duplicated non-TAG genes, and to ~28% of the single genes (see Materials and Methods). Thus, functional assignment is more complete for the *Arabidopsis* data.

**Table 3:**
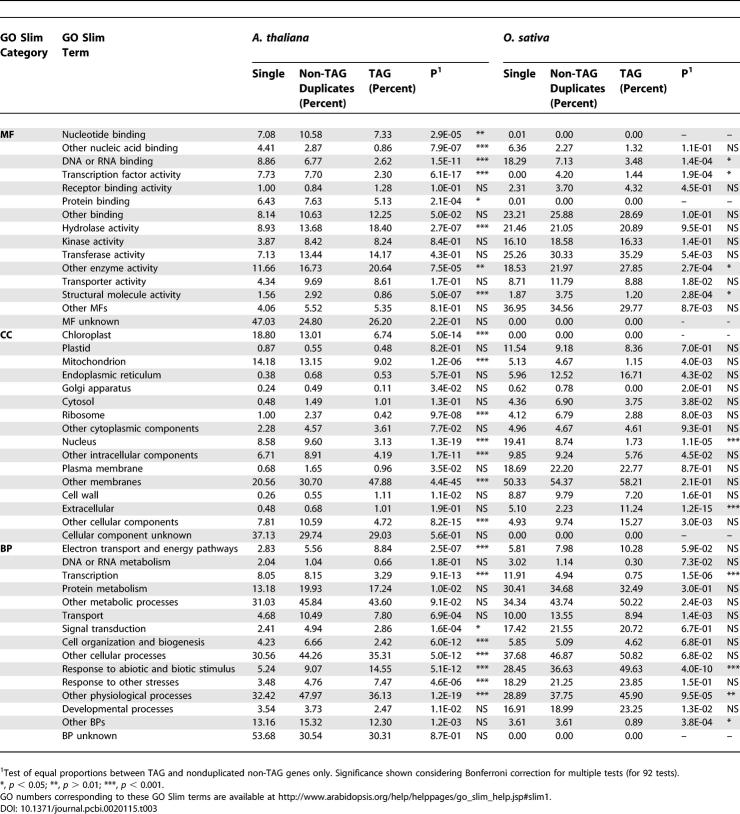
Proportion of TAGs, Duplicated Non-TAGs, and Singletons Genes in GO Slim Categories, Based on the H/0 Dataset

**Figure 5:**
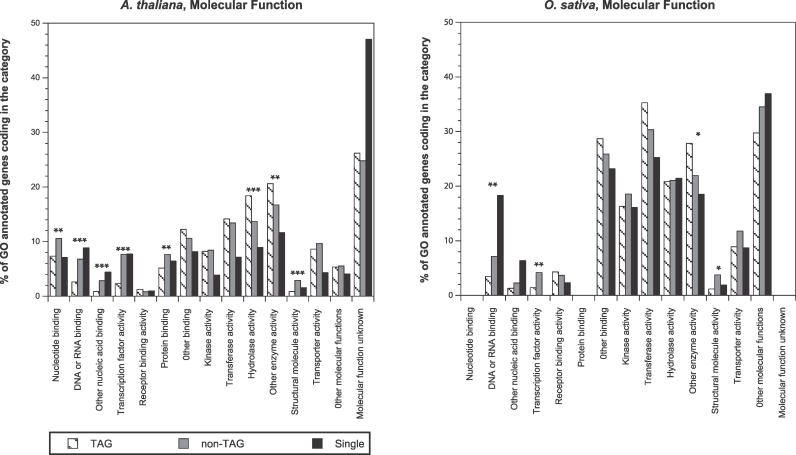
Frequency of Genes in the GO MF categories in *Arabidopsis* and Rice, Based on the H/0 Dataset Only the H/0 datasets are shown. The asterisks above the bars indicate significance of the χ^2^ tests, under the null hypothesis that TAGs and duplicated non-TAG genes have the same proportion. *, *p* < 0.05; **, *p* > 0.01; ***, *p* < 0.001. Bonferonni-corrected for 92 tests.

Numerous differences were evident across the three gene categories (TAG, non-TAG, and singleton; Figure 5, Table 3, Table S5). The results were qualitatively consistent across datasets for A. thaliana but differed somewhat according to the TAG definition for O. sativa. Here, for simplicity, we limit our discussion to results for which there was a significant under- or overrepresentation of TAGs in *Arabidopsis* with a similar, significant (after Bonferroni correction) trend in one of the two rice datasets. Our reasoning is that results consistent across species are more likely to provide general insights into TAG evolution.

In the MF category, there was a relative dearth of TAGs relative to non-TAGs in the “DNA or RNA binding,” “transcription factor activity,” and “structural molecule acitivity” categories. For one of these categories (“DNA or RNA binding”), duplicate genes as a whole are also significantly underrepresented relative to singleton genes, but TAGs are even more underrepresented than duplicate non-TAG genes (Figure 5 and Table 3). In contrast, duplicate genes were overrepresented in the “other enzyme activity” category, with TAGs significantly overrepresented related to duplicate non-TAGs.

Our analyses were hampered by the relatively low number of GO annotated rice genes in the CC and BP GO categories. Nonetheless, for CC functions, TAGs were underrepresented in the nucleus ribosome, mitochondrion, and “other intracellular”components compared with duplicate non-TAGs and singleton genes, but overrepresented for “other membranes” components. For BP functions, TAGs were underrepresented in the “transcription” category relative to both non-TAGs and singletons. In contrast, all duplicate genes (TAGs and non-TAGs) were overrepresented in the “response to biotic and abiotic stimulus” and “electron transport and energy pathways” categories, and TAGs were even more overrepresented relative to duplicated non-TAGs in these categories. The abiotic stress response category includes genes such as those in the mitogen-activated protein kinase pathway (e.g., *MAPKK; MKK1; MEK1*), which are known to process a wide range of external stimuli. The biotic stress category includes, among other things, disease resistance proteins of the TIR–NBS–LRR class.

## Discussion

Arabidopis and rice are predominantly selfing plants with small genomes, but they differ substantially in other aspects. They differ in chromosome number (five versus 12), genome size (the rice genome is ~3.7 larger than that of *Arabidopsis*), polyploid history [22], and growth habit (weedy versus cultivated). They even differ in the timing of their shift from outcrossing to selfing. Rice apparently became a selfer during domestication ~10,000 years ago, but *Arabidopsis* may have become selfing substantially earlier [23]. Their divergence ~150 million years ago [15] makes the two plant lineages as old or older than placental mammals [24].

Despite these differences, our analyses reveal that TAGs in *Arabidopsis* and rice are similar in at least four respects. The first is the proportion of TAGs relative to non-TAG genes. Applying identical homology definitions to both datasets, rice always has a slightly lower proportion of TAGs than *Arabidopsis* (Table 1), but the estimates were similar for the least strict TAG definition (16% for *Arabidopsis* and 14% for rice). These values do not differ substantially from previous reports in the plant literature (~14% to ~18%) [11,16,18], with two exceptions. The first exception is also based on analysis of the finished rice genome. Using a sliding window of fixed length with less stringent homology definitions, the International Rice Genome Sequencing Project reported that up to 29% of rice genes are TAGs [17]. This high proportion does not correlate well with this and other analyses of rice genome sequences. The second exception is maize, where extensive analysis of BAC-end sequences suggests that one-third of maize genes are TAGs [25]. It remains to be seen if the maize genome does contain a substantively larger proportion of genes in tandem arrangement.

The second conserved feature of TAGs is the size distribution of tandem arrays. Both genomes have a preponderance of tandem arrays consisting of only two genes (>62%), with far fewer arrays containing more than three genes (Figure 1). The largest numbers of genes in an array were 20 genes and 33 genes in *Arabidopsis* and rice, respectively, under our least strict (L/10) definitions of TAGs. Using a sliding window approach with unlimited numbers of spacers, the International Rice Genome Sequencing Project identified a putative TAG containing 134 members. Such large clusters are clearly the exception rather than the rule, and do not affect the conclusion that the distribution of TAG size is similar between two plant species that are separated by 150 million years of evolution [15] and differ 3.7-fold in genome size [16,18].

The third conserved feature between species is the distribution of TAG density along chromosomes. In both cases, the TAG density is low in centromeric and pericentromeric regions, even after correcting for the relatively low proportion of non-TAG genes in these regions. For both species, TAG density is positively correlated with recombination, again after correction for the density of non-TAG genes. The correlation coefficients are similar for *Arabidopsis* [11]) and rice (*ρ* = 0.26 to 0.41 depending on TAG definition). Thus, to the extent that *Arabidopsis* and rice are representative, a positive correlation between TAG density and recombination is a general feature of plant genomes. This positive correlation has also been reported in nematodes [26] and may therefore be a general feature of eukaryotic genomes.

The final similarity among the TAGs of *Arabidopsis* and rice relates to functional biases. Caution must be urged interpreting GO-based results, for the following reasons: i) only a relatively small proportion (<50%) of rice genes had GO terms available; ii) there are inherent uncertainties and difficulties with GO definitions, particularly the treatment of paralogs that differ in GO categories, and iii) we emphasized similarities between the two species. Nonetheless, TAGs appear to be underrepresented in nucleic binding functions (i.e., transcription and DNA or RNA binding functions) but overrepresented for extracellular and stress functions. We emphasize that these are TAG-specific biases and not biases due to retention of non-TAG genetic duplicates after a polyploid event. These functional biases have not been noted previously on a genome-wide scale, but analysis of individual gene families have provided circumstantial evidence about such biases, particularly with regard to stress response. For example, genes often found in tandem arrays include NBS–LRR disease resistance genes [27], genes that produce secondary metabolites that function in herbivore resistance [28,29], and glycotransferases that contribute to the ability to cope with environmental challenges [30]. Moreover, a study of 50 *Arabidopsis* gene families showed that the two families with the strongest bias toward tandem organization encoded plant defense functions [31]. Perhaps the most compelling example to date is that of the RLK gene family. Shiu et al. [32] performed a comparative analysis of the receptor-like kinase RLK gene family in rice and *Arabidopsis*. Their analyses revealed an expansion of the RLK family in rice, much of which could be attributed to tandem duplication. RLK genes function in plant growth, development, and defense, but the TAGs were overrepresented for RLK genes that function in plant defense.

### The Pairwise Ks Distribution Differs between Rice and *Arabidopsis*

Despite broad similarities, there are also marked differences between the TAGs of rice and *Arabidopsis*. Blanc and Wolfe [2] originally observed the most striking difference: on average, TAGs in the *Arabidopsis* genome have much lower Ks values than TAGs in the rice genome. Further, as documented previously [2,20], the Ks distribution for TAGs in *Arabidopsis* has a pronounced peak near Ks ~ 0.3 (Figure 4). Haberer et al. [20] and Blanc and Wolfe [2] mention three processes that could generate this peak: i) a recent decline in the rate of generation of tandem arrays, ii) a burst of tandem duplication in the timeframe corresponding to Ks ~ 0.3–0.4, or iii) a recent and increased rate of DNA loss in *Arabidopsis* that preferentially affected TAGs. Blanc and Wolfe [2] favored the last explanation. A final, previously unmentioned, explanation is that there was a substantial increase in homogenizing gene conversion events at the time, corresponding to Ks = 0.3, perhaps as a byproduct of modified patterns of gene conversion during the post-polyploid, diploidization process. None of these mechanisms have been documented, and all of these explanations require further study.

In contrast, the Ks distribution for rice TAGs does not contain an obvious peak [16]. Here it is worthwhile to consider briefly the limitations and pitfalls of pairwise Ks distributions. The evidence for polyploidy in rice is convincing based on collinearity among chromosomes [5], but Ks distributions initially failed to uncover a peak corresponding to a whole genome duplication event [2]. Thus it is clear that Ks distributions lack inferential power and are, at best, inexact tools to infer genomic history [33]. More disturbingly, the number and location of inferred Ks peaks vary dramatically among studies, even when the studies use identical homology definitions [1,2]. In *Arabidopsis,* for example, the peak of the pairwise Ks distribution centers on values as low as 0.45 in one study [1] to as high as 0.8 in others [2,14]. Similarly, studies of the pairwise Ks distribution in rice vary as to the existence of a peak and the Ks value of the peak [2,6,16]. The take-home message is that Ks distributions are remarkably imprecise, wholly dependent on the homology definitions used to identify duplicated genes, and in some cases misleading. Curiously, one of the few robust inferences from studies of Ks distributions in plant genomes has been the peak for *Arabidopsis* TAGs at Ks ~ 0.3. Yet at present it is not clear what evolutionary processes have generated this peak.

### Evolutionary Forces Acting on TAGs

UCO is one of the major mechanisms that generates TAGs. UCO events generate duplicate genes in direct orientation. Tandem arrays can also be produced by intrachromosomal recombination between direct and indirect repeats; these intrachromosomal events tend to produce gene copies in opposite orientation [34]. Our studies reveal that ~80% and ~88% of tandem arrays are in direct orientation in rice and *Arabidopsis,* respectively. These numbers superficially suggest that UCO is the prominent mechanism of TAG generation.

In a study featuring an *Arabidopsis* synthetic tandem array, UCO between sister chromatids generated copy number variants at the rate of ~10^−6^ per plant per F1 meiosis [35]. To put this rate in perspective, the rate of nucleotide substitution is ~10^−8^ or 10^−9^ substitutions per year in plants [36]. Assuming (for simplicity) one generation per year, copy number mutations per TAG are thus three orders of magnitude greater than mutations per nucleotide site. With thousands of TAGs per genome (Table 1), the overall force of mutation is undoubtedly high enough to result in substantial copy number polymorphism among individuals. To date, there is little information about structural and copy number polymorphism in plants (but see [37,38]), but recent experiments have uncovered substantial copy number and structural polymorphism in humans [39].

Because UCO is a function of homologous recombination, the rate of UCO should scale with recombination rates along chromosomes. This leads to the prediction, as yet untested, that copy number polymorphism also scales with recombination rate, purely as a function of the UCO process. A corollary to this prediction is that TAGs should be clustered at higher density in high recombination regions. We have shown that this prediction holds in both *Arabidopsis* and rice, suggesting that mechanisms of tandem duplication are not homogeneous along chromosomes.

Is the distribution of TAGs shaped solely by rates of UCO? This seems highly unlikely, for several reasons. First, the positive correlation between recombination and TAG density is relatively weak. The weak correlation could be a consequence of measurement error for *c,* but it suggests other factors help shape the distribution of tandem arrays. Second, the distribution of array sizes is remarkably consistent between rice and *Arabidopsis,* with a preponderance of arrays consisting of only two genes. Left unchecked, UCO will result in either very large arrays or complete gene loss, leading to rapid gene turnover [40]. In theory, it would be possible to maintain low array size in the absence of selection with a delicate balance between deletion and duplication rates, but under such a model the TAGs should all be relatively new, with low Ks. We see no such Ks bias; in fact, the Ks bias in *Arabidopsis* is toward relatively high (~0.3) Ks (Figure 3). The similarity in array sizes between these two genomes, without an obvious bias to low Ks TAGs, points to relatively strong (and similar) selective forces on array size. Viewed genomewide, it may be accurate to view selection on TAGs as stabilizing, where too few tandem copies (i.e., <1) or too many tandem copies (>2) of most genes are deleterious [11,41].

Yet, the selective forces acting on *individual* tandem arrays must vary substantially. Functional annotations suggest that certain functional classes, such as genes involved in “abiotic stress,” are more likely to be found in tandem than as nontandem duplicates or as singleton genes (Table 3). Similarly, several functional classes, such as “transcription factors” and “DNA binding factors,” are underrepresented as TAGs. It is difficult at this point to assess whether differences among classes (e.g., abiotic stress versus transcription factor) are primarily a function of retention rates, which would be mediated by selection, or duplication rates, which are likely a function of both genomic location (repeat structure, chromatin structure, and recombination rate) and gene structure (some genes, such as LRR genes, may be more prone to tandem duplication). Nonetheless, these results make sense when interpreted in light of selection. Common duplication and retention of “DNA binding factors” or “transcription factors” is liable to affect adversely entire genetic networks, particularly in the absence of commensurate duplication of other members of the pathway. This is one crucial way in which the evolution of tandem duplicates differs from gene duplication via polyploidy. Polyploidy duplicates entire networks, permitting the retention, evolution, and divergence of redundant networks [42]. In contrast, tandem duplication typically copies a single gene and thus a single component of a pathway. Thus, the most evolutionarily successful tandem duplication events are most likely to target genes at the end of biochemical pathways, or genes representing flexible steps in a pathway, where fluctuation in copy number is unlikely to affect downstream genes. It may not be surprising that TAGs are enriched for duplication of membrane proteins and abiotic response genes, as these may provide a rich source for environmental response, often without substantial damage to critical steps in genetic networks.

Duplication by polyploidy is sporadic, and afterward genes are either lost or retained as a duplicated pair. Although the deletion process can be relatively rapid, it seems likely that copy number variation due to polyploidy is not as flexible or variable as copy number derived from tandem duplication. If changes in copy number provide an immediate source for adaptation [43], tandem duplication represents a powerful evolutionary force for plant adaptation. Much discussion has centered on the fact that gene duplication due to polyploidy provides a template for gene neofunctionalization and subfunctionalization [14]. Although not studied in great detail, it is clear that tandemly duplicated genes also diverge in function [28], perhaps to a greater degree than duplicates produced by polyploidy [44] and perhaps biased by gene function [45].

## Materials and Methods

### Protein data.

Predicted rice proteins (version 3.0) were downloaded from The Institute for Genomic Research (TIGR) Web site in September 2005. Version 3.0 consisted of 61,251 putative proteins, corresponding to 57,917 genes, including 14,198 genes annotated as related to transposable elements (TEs). A. thaliana protein sequences were downloaded from NCBI in September 2005. There were 28,860 annotated proteins in the dataset, corresponding to 26,359 genes. For both species, protein sequences were screened for TE using a TBLASTN search [46] against the Plant Repbase September 2005 update [47], with default settings and an E-value cutoff of 1.0. After “merging” (see below), genes encoding proteins with >50% identity to a TE sequence more than >70% of their length were excluded from further analyses. In total, 387 A. thaliana genes and 15,383 O. sativa genes related to TEs were removed.

### Identification of TAGs.

For each species, an all-against-all BLASTP search was performed, using default parameters and an E-value cutoff of 1.0. For each pair of genes, blast-hits were merged to compute the total length and the global similarity of aligned regions. Merging was an iterative process consisting of several steps: i) BLAST hits were sorted according to their E-value, ii) the best hit between two proteins was selected and merged with the next best hit between those two proteins if the overlap between hits was ≤10 amino acids, iii) the process moved to the next best hit, which was merged if it did not overlap with previously selected hits by more than 10 amino acids, and iv) the process was repeated until all blast hits between two proteins were merged. When merging was complete, we calculated the percentage of the protein length aligned and the average percentage of identity over the aligned regions.

After merging BLAST results, two datasets were retained. The first retained protein pairs with ≥30% identity covering ≥70% of protein length. The second set retained protein pairs with ≥50% identity over ≥90% protein length. The two sets were denoted the low stringency (L) dataset and the high stringency (H) dataset, respectively. For both datasets, homologous protein sets, which we call “superfamilies,” were defined by the single-linkage criterion. TAGs were identified as subsets of superfamilies. Genes were defined as TAGs if they belonged to the same superfamily and were either physically adjacent or separated by a prescribed number of nonhomologous intervening “spacer” genes. We varied the definition of TAGs to allow zero, one, five, or ten spacer genes. Superfamily and TAG definitions for rice and *Arabidopsis* are available at http://titus.bio.uci.edu/data.htm.

### Estimation of rice recombination rates.

A contrast between physical and genetic distances was used to estimate recombination rates (*ĉ,* measured in cM/Mb) along rice chromosomes. The average genetic and physical position (on rice virtual chromosomes) of 3,192 BAC clones were downloaded from TIGR (http://www.tigr.org) in December 2005. For each chromosome, we calculated the best fitting polynomial curve between physical and genetic distances data using the R statistical package. Polynomial curves with increasing order were incrementally fit to data from each chromosome, until an additional order polynomial did not significantly improve fit to the data. Recombination rate (*ĉ*) for a physical location on the chromosome was estimated as the derivative of the polynomial. Because centromere positions were not known exactly, we estimated their position from estimates of *ĉ*. If a chromosomal region had *ĉ* = 0.0, this region was denoted the centromere. If *ĉ* > 0.0 along the entire chromosome, the point with lowest *ĉ* was deemed the centromere.

We also estimated recombination by calculating separately for each chromosome arm. To define the arms, we first defined the centromere at the midpoint of the largest group of ordered BACs that showed no variability in genetic distance. We estimated *ĉ* for each chromosome arm with polynomial fitting, as above.

### TAG distribution.

To analyze whether TAGs and genes are clustered along rice chromosomes, we split the chromosomes into 10-kb fragments and coded each fragment as “1” or “0,” depending on whether the fragment did or did not contain a TAG midpoint. We then calculated the Multiple Pool statistic, which detects a non-uniform distribution along the chromosomes [48].

To study TAG density, chromosome sequences were split into 1.0-Mb partitions. Density was calculated for each partition in two ways: i) the number of TAGs divided by the total number of genes, and ii) the number of TAGs divided by the total number of non-TAG genes. The latter calculation of density was used to explore the relationship between recombination rate and TAG distribution in rice in a manner directly analogous to that completed for *Arabidopsis* [11]. The relationship between TAG density and recombination rate was assessed by Spearman rank tests. Telomeric regions were excluded from these analyses, as they appeared to be subject to “boundary effects” for estimation of *c,* but qualitative results did not differ substantially when telomeres were included (unpublished data).

### *Ks* Estimation.

Nucleotide sequence data for *Arabidopsis* and rice were downloaded from NCBI in November 2005 and TIGR in September 2005, respectively. Pairs of homologous proteins within superfamilies were aligned with CLUSTALW [49], using default options, and these alignments were subsequently imposed on the coding region of nucleotide sequences using a PERL script based on Bioperl functions. For both species, we only considered the longest alternatively spliced form of peptides in calculations.

For all pairwise alignments, the Ks substitution rate was calculated using the PAML package and the YN00 program [50]. We only retained pairwise distances for which Ks < 2.0 in analyses.

### GO annotations and categories.

GO annotations were downloaded for *Arabidopsis* and rice from TAIR and TIGR, respectively, in September 2005. We used plant-related GO Slim terms [51], as of 1 April 2005, to explore TAG functions. Each gene can be associated with several GO Slim terms in the MF, CC, and BP GO functional categories. We studied each GO Slim term category independently. For each GO Slim term category, we counted the number of TAGs, the number of duplicated non-TAG genes within a superfamily, and the number of singleton (not a member of a superfamily) genes linked at least once with this GO Slim term category. We determined whether proportions were equivalent among categories (i.e., TAG, non-TAG, and singleton) with Pearson's χ^2^ test. The GO numbers associated with each GO Slim category are listed at http://www.arabidopsis.org/help/helppages/go_slim_help.jsp#slim1.

## Supporting Information

Table S1 Distribution of Arrays' Size, in Percentage of the Total Number of Arrays (32 KB DOC)

Table S2 Partitions and Recombination Rate Estimates for Each Chromosome (70 KB DOC)

Table S3 Spearman Rank Correlations Tests between Recombination Rate and TAG Density for Each Chromosome and Each Dataset (A) Telomeres taken into account. (B) Telomeres removed.
(124 KB DOC)

## Abbreviations

BP: biological process
CC: cellular component
GE: Gene Ontology
Ks: synonymous distance
H: high stringency
L: low stringency
MF: molecular function
TAGs: tandemly arrayed genes
TEs: transposable elements
UCO: unequal crossing over, unequal crossover
