# Supplementary material for: Striking Similarities in the Genomic Distribution of Tandemly Arrayed Genes in Arabidopsis and Rice
Source: PLoS Comput Biol. 2006 Sep 1;2(9):e115. doi: 10.1371/journal.pcbi.0020115 (PMC1557586; doi:10.1371/journal.pcbi.0020115)
Supplement: Supplementary file 1 [file pcbi.0020115.st001.doc]

**Table S1:** Distribution of arrays’ size, in percentage of the total number of arrays

|  | ***A. thaliana*** | | | |  | ***O. sativa*** | | | |
| --- | --- | --- | --- | --- | --- | --- | --- | --- | --- |
|  | **Data set** | | | |  | **Data set** | | | |
| **Array size** | **L/0** | **L/10** | **H/0** | **H/10** |  | **L/0** | **L/10** | **H/0** | **H/10** |
| 2 | 70.6 | 67.9 | 74.6 | 70.6 |  | 76.7 | 62.2 | 79.2 | 68.3 |
| 3 | 18.8 | 17.5 | 17 | 17.1 |  | 14.6 | 18.2 | 14.1 | 18 |
| >3 | 10.6 | 14.6 | 8.3 | 12.3 |  | 8.7 | 19.6 | 6.7 | 13.7 |
| Max Array size | 13 | 20 | 12 | 13 |  | 11 | 33 | 9 | 23 |
