# Supplementary material for: Striking Similarities in the Genomic Distribution of Tandemly Arrayed Genes in Arabidopsis and Rice
Source: PLoS Comput Biol. 2006 Sep 1;2(9):e115. doi: 10.1371/journal.pcbi.0020115 (PMC1557586; doi:10.1371/journal.pcbi.0020115)
Supplement: Supplementary file 2 [file pcbi.0020115.st002.doc]

**Table S2:** Partitions and recombination rate estimates for each chromosome

| **Chr** | **Total Length (bp)** |  | **Region used for recombination analyses “with” telomeres (bp)** | |  | **Region used for recombination analyses “without” telomeres (bp)** | |  | **Estimation of the centromere position (bp)** |  | **Recombination rate (cM/Mb)** | | |
| --- | --- | --- | --- | --- | --- | --- | --- | --- | --- | --- | --- | --- | --- |
|  |  |  | **beging** | **end** |  | **begin** | **end** |  |  |  | **mean**^2^ | **min** | **max** |
| **1** | 43249587 |  | 1640000 | 42650000 |  | 1640000 | 42249587 |  | 16116500 |  | 4.11 | 1.05 | 9.22 |
| **2** | 35876369 |  | 1380000 | 35600000 |  | 1380000 | 34876369 |  | 14247750 |  | 4.43 | 0.19 | 8.92 |
| **3** | 36347804 |  | 1730000 | 35100000 |  | 1730000 | 35100000 |  | 20529700 |  | 4.46 | 1.62 | 7.90 |
| **4** | 35023746 |  | 1750000 | 34020000 |  | 1750000 | 34020000 |  | 12016800 |  | 3.62 | 0.04 | 9.08 |
| **5** | 29695855 |  | 1 | 28760000 |  | 1000000 | 28695855 |  | 11810450 |  | 4.20 | 0.11 | 18.36 |
| **6** | 31198625 |  | 1950000 | 31198625 |  | 1950000 | 30198625 |  | 14719150 - 16152400 |  | 4.03 | 0.00 | 8.49 |
| **7** | 29688601 |  | 620000 | 29688601 |  | 1000000 | 28688601 |  | 9132900 - 11992800 |  | 4.11 | 0.00 | 10.01 |
| **8** | 28309183 |  | 1 | 28309183 |  | 1000000 | 27309183 |  | 13620200 |  | 4.35 | 0.33 | 9.10 |
| **9** | 22680691 |  | 900000^1^ | 22680691 |  | 1000000^1^ | 21680691 |  | 900000 - 4400000 |  | 4.45 | 0.00 | 9.70 |
| **10** | 22698374 |  | 1 | 22698374 |  | 1000000 | 21698374 |  | 8132600 |  | 3.58 | 0.08 | 7.73 |
| **11** | 28369397 |  | 1 | 27480000 |  | 1000000 | 27369397 |  | 12051000 - 13520400 |  | 4.15 | 0.00 | 7.46 |
| **12** | 27492551 |  | 1 | 27492551 |  | 1000000 | 26492551 |  | 11054900 |  | 3.80 | 0.00 | 8.04 |

^1^ begin = 0.9Mb was used in the TAG versus non-TAG distribution analyses relative to centromere/arms regions.

^2^ total mean = 4.12 cM/Mb.
