# Supplementary material for: Striking Similarities in the Genomic Distribution of Tandemly Arrayed Genes in Arabidopsis and Rice
Source: PLoS Comput Biol. 2006 Sep 1;2(9):e115. doi: 10.1371/journal.pcbi.0020115 (PMC1557586; doi:10.1371/journal.pcbi.0020115)
Supplement: Supplementary file 3 [file pcbi.0020115.st003.doc]

**Table S3:** Spearman rank correlations tests between recombination rate and TAG density for each chromosome and each data set (A: telomeres taken into account; B: telomeres removed)

|  |  | **L/0** | | **L/1** | | **L/5** | | **L/10** | | **H/0** | | **H/1** | | **H/5** | | **H/10** | |
| --- | --- | --- | --- | --- | --- | --- | --- | --- | --- | --- | --- | --- | --- | --- | --- | --- | --- |
|  | **Chr** | **rho** | **P** | **rho** | **P** | **rho** | **P** | **rho** | **P** | **rho** | **P** | **rho** | **P** | **rho** | **P** | **rho** | **P** |
|  |  |  |  |  |  |  |  |  |  |  |  |  |  |  |  |  |  |
| **A** | **1** | 0.47 | 0.002 | 0.36 | 0.020 | 0.29 | 0.067 | 0.31 | 0.051 | 0.45 | 0.004 | 0.33 | 0.037 | 0.31 | 0.049 | 0.36 | 0.022 |
|  | **2** | 0.11 | 0.533 | 0.07 | 0.712 | -0.05 | 0.764 | -0.11 | 0.533 | 0.26 | 0.143 | 0.23 | 0.186 | 0.12 | 0.492 | 0.09 | 0.603 |
|  | **3** | 0.44 | 0.011 | 0.45 | 0.010 | 0.39 | 0.027 | 0.36 | 0.042 | 0.27 | 0.130 | 0.31 | 0.082 | 0.29 | 0.107 | 0.22 | 0.208 |
|  | **4** | 0.25 | 0.172 | 0.21 | 0.249 | 0.30 | 0.090 | 0.19 | 0.293 | 0.14 | 0.454 | 0.08 | 0.646 | 0.14 | 0.447 | 0.08 | 0.644 |
|  | **5** | 0.27 | 0.150 | 0.15 | 0.436 | 0.04 | 0.830 | 0.05 | 0.802 | -0.03 | 0.882 | -0.10 | 0.590 | -0.18 | 0.351 | -0.21 | 0.283 |
|  | **6** | 0.56 | 0.002 | 0.53 | 0.004 | 0.26 | 0.178 | 0.20 | 0.285 | 0.34 | 0.071 | 0.40 | 0.033 | 0.31 | 0.107 | 0.31 | 0.099 |
|  | **7** | 0.48 | 0.009 | 0.50 | 0.006 | 0.43 | 0.021 | 0.35 | 0.063 | 0.55 | 0.002 | 0.60 | 0.001 | 0.54 | 0.003 | 0.46 | 0.012 |
|  | **8** | 0.76 | 5.7E-06 | 0.79 | 2.5E-06 | 0.77 | 4.1E-06 | 0.77 | 3.9E-06 | 0.55 | 0.003 | 0.60 | 0.001 | 0.72 | 2.5E-05 | 0.71 | 3.6E-05 |
|  | **9** | 0.43 | 0.046 | 0.36 | 0.099 | 0.39 | 0.075 | 0.38 | 0.079 | 0.22 | 0.326 | 0.15 | 0.493 | 0.19 | 0.405 | 0.26 | 0.246 |
|  | **10** | 0.37 | 0.082 | 0.30 | 0.161 | 0.25 | 0.254 | 0.19 | 0.394 | 0.38 | 0.075 | 0.38 | 0.077 | 0.38 | 0.078 | 0.32 | 0.140 |
|  | **11** | 0.54 | 0.004 | 0.56 | 0.003 | 0.56 | 0.003 | 0.57 | 0.002 | 0.35 | 0.077 | 0.33 | 0.096 | 0.43 | 0.026 | 0.49 | 0.010 |
|  | **12** | 0.41 | 0.035 | 0.39 | 0.043 | 0.32 | 0.109 | 0.38 | 0.054 | 0.04 | 0.855 | 0.21 | 0.301 | 0.31 | 0.114 | 0.35 | 0.076 |
|  |  |  |  |  |  |  |  |  |  |  |  |  |  |  |  |  |  |
| **B** | **1** | 0.48 | 0.002 | 0.37 | 0.016 | 0.31 | 0.052 | 0.33 | 0.039 | 0.45 | 0.004 | 0.34 | 0.031 | 0.31 | 0.046 | 0.36 | 0.021 |
|  | **2** | 0.10 | 0.594 | 0.02 | 0.902 | -0.05 | 0.788 | -0.10 | 0.574 | 0.22 | 0.220 | 0.21 | 0.249 | 0.09 | 0.608 | 0.07 | 0.683 |
|  | **3** | 0.44 | 0.011 | 0.45 | 0.010 | 0.39 | 0.027 | 0.36 | 0.042 | 0.27 | 0.130 | 0.31 | 0.082 | 0.29 | 0.107 | 0.22 | 0.208 |
|  | **4** | 0.25 | 0.172 | 0.21 | 0.249 | 0.30 | 0.090 | 0.19 | 0.293 | 0.14 | 0.454 | 0.08 | 0.646 | 0.14 | 0.447 | 0.08 | 0.644 |
|  | **5** | 0.27 | 0.158 | 0.17 | 0.376 | 0.11 | 0.564 | 0.10 | 0.596 | -0.03 | 0.874 | -0.07 | 0.736 | -0.13 | 0.500 | -0.17 | 0.379 |
|  | **6** | 0.57 | 0.002 | 0.55 | 0.003 | 0.25 | 0.202 | 0.20 | 0.303 | 0.34 | 0.079 | 0.40 | 0.036 | 0.29 | 0.130 | 0.32 | 0.101 |
|  | **7** | 0.49 | 0.009 | 0.46 | 0.015 | 0.44 | 0.020 | 0.36 | 0.057 | 0.52 | 0.005 | 0.53 | 0.004 | 0.46 | 0.015 | 0.37 | 0.050 |
|  | **8** | 0.75 | 1.8E-05 | 0.78 | 4.9E-06 | 0.77 | 8.4E-06 | 0.77 | 8.2E-06 | 0.50 | 0.010 | 0.57 | 0.003 | 0.70 | 1.0E-04 | 0.69 | 1.3E-04 |
|  | **9** | 0.38 | 0.089 | 0.30 | 0.192 | 0.32 | 0.153 | 0.30 | 0.183 | 0.11 | 0.625 | 0.05 | 0.824 | 0.11 | 0.629 | 0.18 | 0.442 |
|  | **10** | 0.55 | 0.011 | 0.55 | 0.011 | 0.50 | 0.023 | 0.49 | 0.026 | 0.56 | 0.009 | 0.57 | 0.008 | 0.52 | 0.016 | 0.51 | 0.020 |
|  | **11** | 0.50 | 0.011 | 0.54 | 0.005 | 0.54 | 0.005 | 0.55 | 0.004 | 0.32 | 0.114 | 0.32 | 0.107 | 0.42 | 0.031 | 0.47 | 0.017 |
|  | **12** | 0.42 | 0.040 | 0.38 | 0.061 | 0.30 | 0.143 | 0.37 | 0.072 | 0.00 | 0.988 | 0.20 | 0.332 | 0.29 | 0.161 | 0.33 | 0.110 |

Significance level: *: P<0.05; **: P<0.01, ***: p<0.001 (with Bonferroni correction for 12 tests)

Note : for Bonferroni corrections: 0.05/12=0.0042; 0.01/12=8.3E-04; 0.001/12=8.3E-05)
